# Ratings and experiences in using a mobile application to increase physical activity among university students: implications for future design

**DOI:** 10.1007/s10209-022-00962-z

**Published:** 2023-01-05

**Authors:** Caroline A. Figueroa, Laura Gomez-Pathak, Imran Khan, Joseph Jay Williams, Courtney R. Lyles, Adrian Aguilera

**Affiliations:** 1 School of Social Welfare, University of California, 102 Haviland Hall, Berkeley, CA 94720-7400 USA; 2 Department of Computer Science, University of Toronto, Toronto, Canada; 3 Zuckerberg San Francisco General Hospital, University of California, San Francisco, CA USA; 4 Technology, Policy, and Management, Delft Technical University, Delft, The Netherlands

**Keywords:** Exercise, Telemedicine, Students, Mental health, Attitude

## Abstract

University students have low levels of physical activity and are at risk of mental health disorders. Mobile apps to encourage physical activity can help students, who are frequent smartphone-users, to improve their physical and mental health. Here we report students’ qualitative feedback on a physical activity smartphone app with motivational text messaging. We provide recommendations for the design of future apps. 103 students used the app for 6 weeks in the context of a clinical trial (NCT04440553) and answered open-ended questions before the start of the study and at follow-up. A subsample (*n* = 39) provided additional feedback via text message, and a phone interview (*n* = 8). Questions focused on the perceived encouragement and support by the app, text messaging content, and recommendations for future applications. We analyzed all transcripts for emerging themes using qualitative coding in Dedoose. The majority of participants were female (69.9%), Asian or Pacific Islander (53.4%), with a mean age of 20.2 years, and 63% had elevated depressive symptoms. 26% felt encouraged or neutral toward the app motivating them to be more physically active. Participants liked messages on physical activity benefits on (mental) health, encouraging them to complete their goal, and feedback on their activity. Participants disliked messages that did not match their motivations for physical activity and their daily context (e.g., time, weekday, stress). Physical activity apps for students should be adapted to their motivations, changing daily context, and mental health issues. Feedback from this sample suggests a key to effectiveness is finding effective ways to personalize digital interventions.

## Introduction

Physical inactivity is a major global health problem. Almost half of American adults and 75% of adolescents do not adhere to the minimum recommended physical activity guidelines [1]. Insufficient physical activity is associated with a higher risk of developing chronic diseases and mental illness, and is estimated to cause around 8% of deaths worldwide [2].

A focus on prevention, including stress management and physical activity promotion, could directly benefit individuals’ physical and mental health and might also prevent the future onset of mental disorders [3–5]. Increasing physical activity also has other health benefits including preventing chronic disease and obesity [6] and ameliorating symptoms of depression and anxiety [4, 7]. Physical activity may also benefit mental health during trauma and uncertainty. For instance, in college students, increasing the minutes of physical activity during COVID-19 stay at home orders was associated with increased positive affect [8].

University study is associated with a decline in physical activity [9]. A meta-analysis showed that a considerable proportion of university students engage in higher levels of sedentary time compared to the general young adult population [10]. Low levels of physical inactivity and high sedentary time have been associated with increased psychological distress in students [11].

Students are also a population a priori at risk of mental health disorders [12, 13]. A national survey including more than 450.000 students from 2009 to 2015 showed that self-reported diagnoses and treatment of several mental health conditions are increasing among college students [14]. Furthermore, an estimated 50–80% of college students who struggle with mental health issues do not seek treatment, with even lower numbers among ethnic minorities, males and LGBTQ students. Students in ‘high achieving’ schools may be at a particularly high risk [15]. Preventative smartphone interventions could help reduce some of the strain on college counseling centers, which serve a growing number of students [14].

Persuasive technology, i.e., interactive applications or systems designed to promote, change, or reinforce desired behavior, has been used as a powerful tool to support physical activity change [16, 17]. A systematic review spanning 15 years of research (80 papers) on persuasive technologies for promoting PA found that mobile phone-based PTs are effective tools; 79% of reviewed studies reported successful or partially successful outcomes, especially when combined with tracking/monitoring or behavioral theories [18]. There is a wealth of mobile apps and interventions designed to help people engage in physical activity [19–21], including apps for university students [22, 23], who use their smartphones for the majority of their waking hours [24].

Text messaging is low-cost, widely used, and convenient, as participants do not need to open the app to see messages [25]. Using texting can enhance behavioral interventions as it allows for in-the-moment, personally tailored notifications [25]. Surprisingly few studies have examined students’ opinions of the content of physical activity mobile apps using text messaging. A systematic review of PA studies (website-based or face-to-face) in university students found that most studies did not specify opinions the content of the information (e.g., about benefits of PA, suggestions to adopt and maintain PA) provided to participants [26]. This makes it difficult to understand effective components of PA interventions. As argued by Klasjna et al., understanding of users’ experiences with behavior change strategies embedded in systems (here a mobile app) is key to promoting behavior change [27]. Thus, examining feedback on the content of PA text messaging interventions can provide valuable lessons for the design of future texting interventions for university students.

We conducted a 6-week study of a physical activity mobile application, called ‘DIAMANTE’ in a sample of undergraduate and graduate students enrolled at the University of California, Berkeley. DIAMANTE is designed to encourage individuals to increase their physical activity through motivational text messaging and step tracking by participants’ pedometer in their personal smartphones. Different types of motivational messages were sent randomly to participants daily.

With this study, we add to existing mostly formative work examining text messages for physical activity promotion by providing a mixed-method analysis of university students’ opinions on the content of text messages for increasing physical activity after using an app for 6 weeks. We report students’ qualitative and quantitative feedback on the mobile app, text messaging content and their preferences for future interventions, and identify and discuss important feedback themes. Given the at-risk status of this population for mental health disorders, in exploratory analyses we also examine differences in quantitative feedback and reported themes by students’ depression-risk status at the start of the study. Taken together, this study will provide knowledge on how to design effective text messaging for physical activity promotion in university students.

## Methods

### Study setting and participants

This is a secondary data-analysis of a clinical trial. The study methods were reported previously in more detail [28]. A total of 103 currently enrolled undergraduate and graduate students were recruited from the University of California, Berkeley from 12/9/2019 until 25/10/2019 through the Social and Experimental Research Lab (Xlab) app. Students that did not have a smartphone were not able to exercise due to disability, or had plans to leave the country during the 6-week study were not eligible to participate. Participants used a mobile phone app, “DIAMANTE” developed by Audacious Software (available at https://diamante.healthysms.org/) for six weeks. This application tracks step counts by pooling from Google Fit, Apple HealthKit or the built-in pedometer on patients’ phones. We also employed a text messaging platform HealthySMS, developed by Audacious software and the authors, to send text messages and manage patient responses back to our system. The app is freely available to download from the Apple App Store and Android Google Play application.

### Text messages

Participants received 2 messages per day (one feedback and one motivational), sent one minute apart for six weeks. We adapted a text messaging bank originally designed for a clinical population [29] by removing messages about chronic disease or family, and adding messages about the benefits of walking on brain health and concentration, and walking with friends. Motivational text messages were designed according to a behavioral theory framework, using the Capability, Opportunity, Motivation, Behavior (COM-B) model (12 messages in every category) [30, 31]. COM-B is a behavioral change model that proposes that engaging in a particular behavior depends on the dimensions of capability (physical and psychological), opportunity (social and physical) and motivation (emotions, beliefs, and self-efficacy needed to engage in the behavior). Messages were designed to fit into these three dimensions. Further, about half the messages were framed with a social connotation (i.e., exercising with friend or being healthy for others), and half were individually framed (exercising for yourself). Participants also received one feedback message daily, with information on their step count and step goal in the previous day. We included feedback messages that were positively framed (e.g., you can do it!) and negatively framed (e.g., you can do better!). Participants received different types of text messages (Tables 1, 2) at varying times during the day.

**Table 1:** Examples of messages in Feedback category

Feedback categories	Examples
1. Reaching goal	Yesterday, you did not reach your goal
2. Steps walked yesterday	Yesterday, you walked 3824 steps
3. Walked more/less today than yesterday	Yesterday, you walked more than your goal
4. Steps walked yesterday, plus a positive/negative motivational message	You walked 8000 steps yesterday. Great job!

**Table 2:** Examples of messages in Motivational category

Motivational categories	Examples
1. Capability	Push yourself a bit further with the help of friends. They believe in you!
	Believing in yourself is the first step towards reaching your goal
2. Motivation	Going for a walk can improve your mood and clear your mind
	A recent study showed that brisk walking for just 5 min produces surprising improvements in thinking speed and spatial awareness. Wow!
3. Opportunity	Find 30 min in your day to go for a walk. That is less time than it takes to watch one episode of a TV show
	Is there a local park you’ve been wanting to visit with your friends? Use it as an opportunity to get out and walk more steps!

Different types of motivational messages were sent randomly to participants daily (i.e., equal probability of receiving different types of messages). A sub-group (*n* = 23) received text messages chosen by a Thompson sampling (TS) algorithm a type of machine learning, to personalize the type and frequency of messaging [32]. In a post hoc analysis, we examined these differences for the random versus TS messaging group.

### Measures

#### Quantitative and qualitative questions baseline

A questionnaire was administered at baseline that included questions about demographics, socioeconomic status, baseline health and physical activity. To measure depressive symptoms, participants filled in the eight-item Patient Health Questionnaire depression scale (PHQ-8), a validated self-report questionnaire measuring depressive symptomatology [33]. Anxiety symptoms were measured the General Anxiety Disorder scale (GAD-7) [34]. In the baseline questionnaire, we included open-ended questions asking what different situations, times of day, or factors in their life that might affect what kind of text messages they would find useful to motivate them to exercise. Participants received $15 USD for their participation in the baseline visit.

#### Qualitative feedback via text message and calling

About 3 weeks into the study, participants were asked to provide their opinions on the app via text message via an automated text message sent by our system. In addition, we asked participants via text message whether we could call them for a short follow-up phone interview on their opinion of the app. The follow-up phone interview included questions on what participants’ barriers and motivations to exercise, and what they liked and disliked about the app content. Participants did not receive any financial compensation for their texting feedback or participation in the phone interview.

#### Quantitative and qualitative feedback exit

At six-week follow-up, participants were invited for an online remote exit interview on their personal digital devices. We asked participants to which degree, in multiple-choice format, they were satisfied by the amount and timing of the text messages, and to what extent the messages made them feel encouraged and supported. The exit questionnaire also asked for feedback about the app in open question format. Questions included: which messages participants found particularly motivating, or de-motivating, times or situations that the texts where helpful or unhelpful, and if they noted any changes in their health by using our system. Finally, we asked participants which kinds of messages would motivate them to become more active. Participants received $25 USD for completing the exit interview.

### Analysis

We applied a constructivist grounded theory approach [35] to our qualitative analysis. LGP developed an initial codebook, which CAF used to code a subset of transcripts. We also employed open coding to generate codes inductively. After all co-authors met to discuss emerging themes, we revised our codebook to incorporate inductively and deductively derived codes that captured user feedback and attitudes related to the content and physical activity motivators. The transcripts were coded independently by CAF and LGP using Dedoose, a qualitative software program [36]. Our results derive from data tagged with codes centered on physical health, mental health, context, positive content, negative content, motivation for exercise, and technical aspects/design of the app. We memoed on the data tagged with these codes in two rounds of analysis to generate the themes we present in this paper. We used Rstudio Version 1.2.5033 for quantitative analysis. Quantitative feedback between participants in the TS group and the uniform random group (post hoc analysis) were compared using Chi-square and Fisher’s exact tests as appropriate.

### Data availability statement

Anonymized data are available as available on request.

## Results

### Participants

In total, 103 participants enrolled in the study downloaded the app and provided baseline interview data. Of these participants, 7 never received the daily text messages and were therefore not included in texting feedback or the exit questionnaire data. Three participants dropped out during the study by texting ‘STOP’ to our system. These data were included in this analysis.

#### Demographic characteristics

The majority of the participants were female (69.9%) with a mean age of 20.2 (2.50) years; more than half of the sample identified as Asian or Pacific Islander (53.4%); half (52.2%) indicated that they engaged in regular physical activity in the last 6 months; 63% of the sample had elevated depressive symptoms. Table 3 provides the baseline demographic characteristics.

**Table 3:** Characteristics baseline

	Overall
	(*N* = 103)
*Age (years)*	
Mean (SD)	20.2 (2.50)
*Self-reported ethnicity*	
Asian or Pacific Islander	55 (53.4%)
Hispanic/Latino(a)	13 (12.6%)
Multi-ethnic	12 (11.6%)
Refused	3 (2.9%)
White or Caucasian	20 (19.4%)
Born in the US (Yes)	60(58.3%)
*Physical activity*	
Regular physical activity last 6 months (Yes)	54 (52.4%)
*Do you want to be more physically active?*	
No	1 (1.0%)
Not sure	5 (4.9%)
Yes	97 (94.2%)
Self-reported minutes of moderate/vigorous exercise per week (IPAQ)	
Median [Min, Max]	150 [0.00, 840]
*Psychological questionnaires*	
PHQ-8 (depressive symptoms) Mean (SD)	5.93 (3.93)
Elevated depressive symptoms (PHQ-8 ≥ 5)	63%
GAD-7 (general anxiety) Mean (SD)	5.09 (4.97)
Elevated anxiety symptoms (GAD-7 > 5)	41.7%

*PHQ-8* Patient Health Questionnaire-8, *IPAQ* International Physical Activity Questionnaire, *GAD-7* General Anxiety Depression Scale-7. A PHQ-8 score above 5 is considered at risk for Major Depressive Disorder [37]. A GAD-7 score above 5 is indicative for at risk for anxiety disorder [34]

### Texting preferences

The majority of the sample indicated that they preferred texting as a communication method (57.3%) as opposed to calling, send several text messages per day (80.2%) and use apps on their phone all the time (40.8%) or several times an hour (34%). The majority of the sample was already tracking their steps in some way (58.3%), and about half of participants allowed a mobile application to track their location all the time (49%). The majority (80.2%) used iPhones.

### Quantitative feedback at exit

The majority of participants reported that the number of messages they received was just right (60.5%). Most wanted to receive one text message per day (29.1%). Participants widely differed in times that they preferred receiving messages, with late morning (9 am–12 pm) being the most common answer (22.1%). The most common reason (47.7%) for the messages arriving at an inconvenient time was participants not carrying their phone. 54.7% felt that the text messages encouraged them to be physically active, or felt neutral toward the intervention, and 45.4% disagreed or strongly disagreed. 64% felt supported by the messages or neutral (see Table 4 for details). There were no differences in participants feeling encouraged to exercise by the messages (*p* = 0.54, two-sided), or supported by them (*p* = 0.46, two-sided) for the TS versus the random messaging group (post-hoc analysis).

**Table 4:** Quantitative feedback on the app at Exit Questionnaire

	Total (*n* = 87)
*App encouraged Physical Activity*	
Agree	22 (25.6%)
Disagree	27 (31.4%)
Neither agree nor disagree	25 (29.1%)
Strongly disagree	12 (14.0%)
*Felt support by the app*	
Agree	30 (34.9%)
Disagree	22 (25.6%)
Neither agree nor disagree	25 (29.1%)
Strongly agree	2 (2.3%)
Strongly disagree	7 (8.1%)
*End amount of messages received was:*	
Far too few	1 (1.2%)
Far too many	6 (7.0%)
Just right	52 (60.5%)
Too few	5 (5.8%)
Too many	22 (25.6%)
*Preferred amount of messages:*	
1/day	25 (29.1%)
2–3/day	4 (4.7%)
2–3/week	22 (25.6%)
4–6/week	18 (20.9%)
None	3 (3.5%)
Once/week	14 (16.3%)

### Qualitative feedback

Baseline: 103 participants provided written responses to open questions during the baseline interview. 36 participants provided their opinions via text message. Eight of these participated in a 10-min follow-up interview by phone. 86 participants provided responses to open-ended questions in the exit interview.

**Fig. 1:**
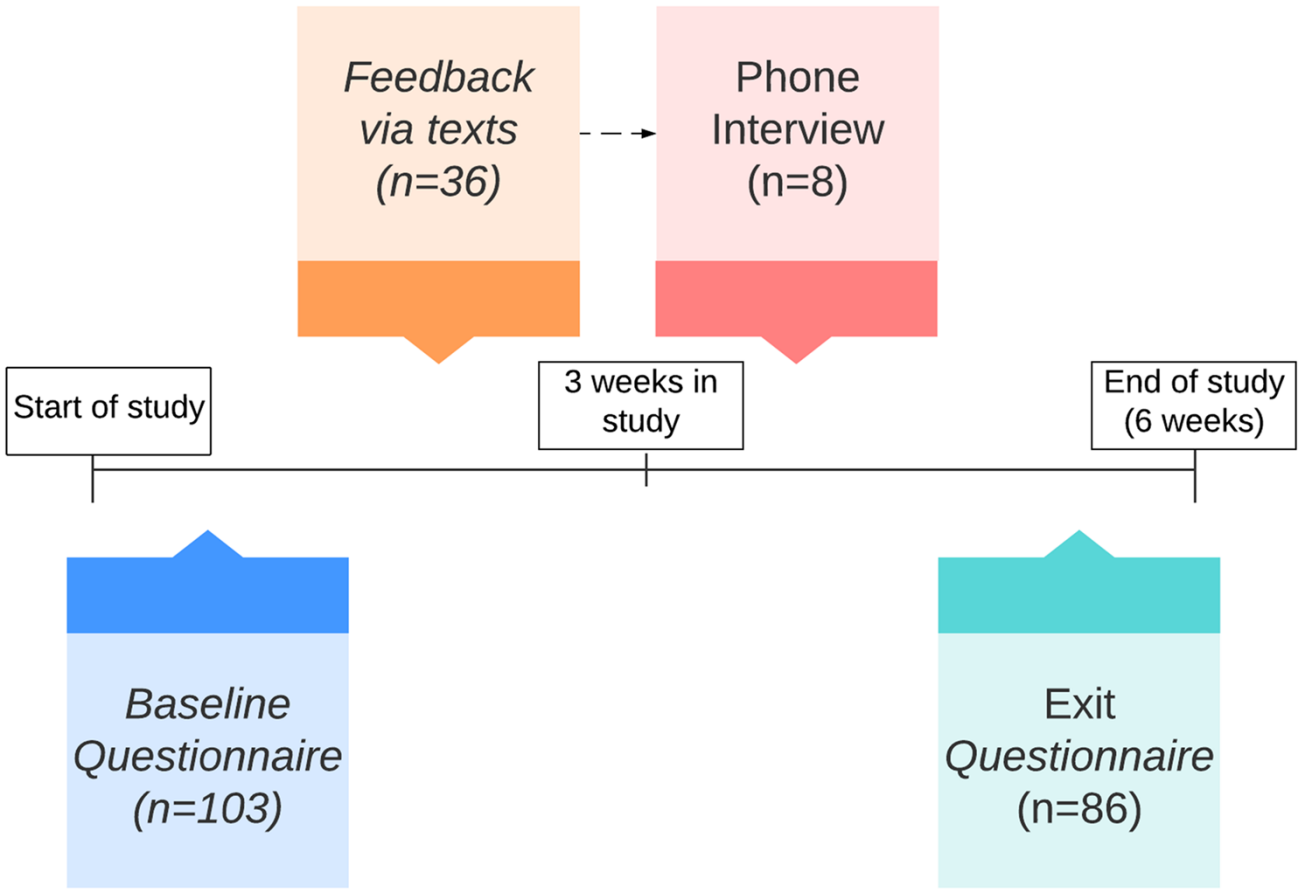
Different phases of feedback during the 6-week study. The baseline questionnaire (*N* = 103) was administered at the start of the study. Participants were asked to give their feedback on the app 3 weeks into the study (*N* = 36). A subset of the participants that gave feedback via the app also participated in a phone interview (*N* = 8). Participants filled in the exit questionnaire at the end of the study (*N* = 86)

Figure 1 shows an overview of the different feedback phases and the number of participants.

We discarded responses that only had single word answers (e.g., yes, no, not sure). This led to the removal of data of 2 participants being excluded. In total, we coded 880 excerpts belonging to 101 participants. Below, we divide the feedback subsections into positive/helpful messages and negative/annoying messages. Within these subsections, we divide the feedback into different subthemes, identified through grounded theory.

#### Feedback on text messaging to encourage physical activity

Throughout the qualitative data collection, we asked participants to come up with messages they would prefer to receive, and under which conditions, texting would or would not be helpful in encouraging them be more physically active. Additionally, we asked which messages participants found particularly motivating, and which were de-motivating or unhelpful.

##### Helpful, positive messages

The messages that participants liked or wanted to receive were structured around (1) physical and mental health benefits of exercise, (2) positive framing, and (3) quantitative/concrete feedback.

First, participants found messages about the benefits of exercise for mental and physical health motivational. For example:

“It is a positive opportunity, because it shows that you can really beneficially impact your health just by walking. I keep it on my mind and it has lead to more walking.”

Another participant provided feedback on a specific message while reflecting on the impact of exercise in their mental health:

“I personally really liked the one ‘exercise is as effective for treating depression and anxiety as therapy’. I have been through depression, but I did not know back then that exercise would help release the negative emotions. This message reminded me of that time period and really made me realize the activities I was involved actually helped me so much in overcoming depression, unconsciously.”

Some mentioned the possibility of specific support for anxiety and depression:

“Partner up with psychologists at UC Berkeley for them to suggest this app to students with depression and anxiety”

Further, participants reported liking messages that were framed positively and reflected encouragement*,* writing comments like:

“I liked the messages that were more motivational or focused on positive parts of exercising such as doing it with friends or that I am almost there and that I should keep pushing.”

Some participants also mentioned liking messages that provided concrete facts about their exercise behavior: *“I liked the messages that had quantitative info the most. Giving hard numbers made it tangible.”*

##### Negative, annoying messages

We divided the negative feedback into (1) the content was not context dependent, (2) the content did not match motivators to physical activity, (3) messages were discouraging.

The greatest source of negative feedback about the messaging content was its lack of personalization and contextualization. This is expressed in the following example:

“[W]hen I'm busy and in the middle of something (which is most of the time during the day - 9am-5pm. and some nights I have meetings 5-7pm and don't like my phone going off during the meeting). But the texts that were most annoying and unhelpful are the ones that were telling me "you can do better!" without any indication for HOW I could do better and logistically fit this into my schedule.”

Or:

“When I'm going through a depressive episode, I'm way less likely to respond to a message that asks me to simply exercise. Vs if it's a "normal" day, a simple message might have more of an effect.”

The most often mentioned foci of personalization were timing and day(s) of the week and stress/business, though these varied substantially from person to person.

Further, though some students mentioned weight loss or physical appearance as motivation to exercise, some disliked messages that mentioned weight loss goals and other extrinsic motivators to exercise. For example, a student dismissed phrases such as “You can start looking great!” as being “*[a] little bit triggering… I’d prefer if the texts would be only about health improvement and not society beauty standards.*”

In addition, some students tagged messages making references to family members and loved ones as being especially annoying, whereas others liked these messages.

Several students also expressed disappointment about not receiving more practical tips for exercise and for engaging in other kinds of exercise beyond walking. One participant, for example, suggested adding concrete cues to other forms of exercise:

“I would find great to have a way to incorporate other gym activities. For example I have been biking since a few days, so my steps have decreased but I am more active.”

The second major negative piece of feedback related the messaging content was that some of the messages were discouraging. Some respondents expressed dislike for the “condescending” nature of certain messages that made them feel discouraged, “called out” or “guilty” or that came off as “scolding” or “punitive.” Here is a particularly evocative excerpt:

“I don't like those discouraging ones, such as ‘you walked less than your goal,’ ‘you did not achieve your goal’. I probably set a goal which was hard to achieve, so I did not achieve the goal most of the days. And it has been kind of annoying and stressed to receive such messages frequently.”

### Exploratory post hoc analysis: differences in frequency of themes for depression status

Because many participants had elevated depression scores and mental health had a prominent role in participant feedback, we examined the differences in coded mental health themes over all prompts for feedback by participants’ depression profiles (high/low depressive symptoms) in an exploratory analysis. We examined whether participants talked about their mental health (e.g., mood, depression, stress) in their feedback (i.e., mental health feedback present vs. absent). Overall, participants with elevated depressive symptoms (PHQ-8 > 5) more often mentioned mental health (57% of participants versus 33%, *X*^2^ = 4.26, *p* = 0.039), either as a benefit of exercising more, or as a barrier to exercise (e.g., being too stressed or depressed). There were no differences in the mentioning of physical health (57% vs. 64%, *X*^2^ = 0.22, *p* = 0.63).

## Discussion

We analyzed feedback from university students during a 6-week physical activity text messaging study. Overall, participants’ opinions about the texting system were very mixed. Qualitative data indicated that participants liked messages on the physical and mental health benefits of exercise, positively framed content and concrete feedback on their steps. Participants disliked messages that did not match their motivations to physical activity, were not context dependent, or had a discouraging tone.

### Relation to previous work

Previous work has long acknowledged that persuasive technologies for physical activity need to accommodate the individual’s changing needs [38]. A systematic review of 80 articles on persuasive technology for physical activity also found that personalization is among the top 10 most effective strategies for behavior change [18]. Oinas-Kukkonen and harjumaa (2008) also argued in their persuasive design model that tailoring and personalization (offering argument/content most likely to be relevant for the user) are important design strategies in supporting users to carry out the primary task (in this case physical activity) [16]. Work by Munson and Consolvo [39] suggested that reminders should be combined with context awareness to deliver them when the user is likely to be in a position to perform an activity. A study in university students, Middelweerd and colleagues, found that students felt annoyed by reminders to engage in physical activity when delivered at inconvenient times (e.g., after recent PA or late in the evening) [40]. Here we also found that most participants preferred messages adapted to their daily circumstances.

### Recommendations

Based on our results and previous work, we provide four recommendations for the development of physical activity text messaging interventions for student populations.

(i) Messages should adapt to individuals’ changing circumstances;
  Participants mentioned messages should be tailored to their previous physical activity (e.g., a different message if they just exercised versus sat still), their mood/stress, the time of day, and weekday. They indicated that messages were not effective when delivered at inconvenient times, for instance, during a university class.
  Our findings confirm an important role for ‘Just-in-Time-Adaptive-Interventions (JITAIs), which alter message delivery based on these ‘contextual’ factors [41]. In a qualitative study, participants perceived just-in time messages more positively than non-context aware messages [42]. However, JITAIs have challenges. For instance, sensing psychological or affective states through self-report or physiological measures is difficult, and few studies have attempted to do so [43]. Future work should identify which contextual variables may best predict physical activity behavior, and how these variables change over time. This would help us understand when, and in what circumstances; text messages are most effective.
(ii) Feedback on, and suggestions for, physical activity should be concrete and varied;
  Participants preferred detailed and encouraging feedback on their steps and physical activity. They also wanted the text messages to give more detailed ideas about exercise activities besides walking. This is technically more challenging. Activities including running, biking, or visiting a gym are difficult to measure by the in-built phone accelerometer. The app could automatically log various activities using individual’s geolocation combined with a physical activity diary. However, geolocation tracking leads to high battery usage and additional security risks [44]. Sampling geolocation at a lower rate (e.g., every 10 instead of 2 min) might preserve battery life while maintaining high accuracy, though this needs to be confirmed in wide-scale studies [45].
(iii) Those with mental health symptoms may need a more tailored system;
  Almost half of participants in this study (47%) mentioned mental health in their feedback. In an exploratory analysis, we observed that those with high depression scores did so more often. Participants mentioned mental health as a motivation to be more physically active, but also as a barrier to exercise. Many were positive about messages describing the benefits of exercise on mental health. However, participants also noted that text messages might be less effective when they feel stressed or down. Increasing evidence shows that a dual approach of maximizing fitness while improving affective experiences might work particularly well for depression [46]. Mobile physical activity interventions might be more effective if text messages could tailor to participants’ affect, or if specific mental health modules are incorporated in the application. Of note, we observed elevated depression scores in 63% of participants. This suggests a high need for interventions that also targets mental health symptoms in students.
(iv) Messages should be personalized to the physical activity motivations and barriers of the user;
  In line with previous literature [38], one of the most important lessons from this study is the limitation of a one-size-fits-all physical activity text messaging system for university students. Participants gave varied feedback on the intervention as a whole, and on which individual messages they found motivational. For instance, some mentioned socially oriented messages as motivational, whereas others, who felt more internally motivated, disliked these messages. This corroborates the findings by Yan et al. [47] that depending on an individual’s social support needs, SMS that assumes positive reinforcement from family and friends may fail to promote physical activity.
  Researchers could base messages on participant personality or motivational profile. For instance, previous work indicates that people’s personality traits such as Openness, Extraversion, and Agreeableness are associated with how they rate the motivational level of behavior change theory text messages [48]. Further, some participants commented that messages referring to looking attractive through physical activity reinforce potentially harmful societal standards. When tailoring messages, researchers should not only contemplate effectiveness, but also the potentially negative implications of this type of messaging. This may particularly harm participants with, for example, eating or compulsive exercise disorders.
  Machine learning methods can also be used to personalize mobile health, and can be combined with a JITAI to select the best real-time treatment policy (e.g., activity suggestion or motivational message) [41, 43]. Here, we used Thompson sampling, a type of machine learning, in a subset of participants to select messages deemed most effective for individual users on a daily basis. However, we did not find differences in subjects’ perceived motivation by the messages. The length of this study or the number of participants might not have been sufficient to find effects. To date, mobile health studies using machine learning have shown some promising effects, but the number of studies is still too small to for a rigorous evaluation of the machine learning methods [49].

### Limitations

We employed convenience sampling of university students. The students in this sample were not selected on their baseline physical activity, or their desire to become more physically active. This may have led to a selection of students less motivated to increase their number of daily steps and thereby to lower engagement/more critical evaluation of the app. Further, the percentage of students answering feedback questions over text message (*n* = 36) and semi-structured interviews (*n* = 8) may be a selective (e.g., more engaged) sample. Finally, the text messages were originally designed for a clinical population. Though we adapted and edited the messages, this could also have led to more negative feedback and lower engagement.

### Strengths

Most mobile health studies do not report detailed participant perspectives and opinions on the specific content of physical activity applications. Here, we collect detailed quantitative and qualitative feedback and analyze students' perceptions to provide concrete recommendations for designing student digital physical activity interventions. In comparison with previous work, we studied a relatively large and diverse sample of students. Further, we explore the relationship between opinions on physical activity apps and depression symptoms in university students, which to our knowledge has not been assessed by previous studies. By examining students’ opinions during the use of the system we can increase our understanding of the effective components of text-message-based physical activity interventions. The results of this study can be used to maximize the effectiveness of future physical activity interventions for student populations.

## Conclusion

Digital interventions can benefit from going beyond a one-size-fits-all approach. Student physical activity text messaging systems should: (1) be personalized/contextualized; (2) provide concrete and varied feedback; (3) incorporate mental health support; and (4) adapt to the user's physical activity barriers/motivations. University students are an at-risk sample for physical inactivity and mental health disorders. More digital interventions need to be designed for this population.
